# Neuronal leucine-rich repeat 1 negatively regulates anaplastic lymphoma kinase in neuroblastoma

**DOI:** 10.1038/srep32682

**Published:** 2016-09-08

**Authors:** Shunpei Satoh, Atsushi Takatori, Atsushi Ogura, Kenichi Kohashi, Ryota Souzaki, Yoshiaki Kinoshita, Tomoaki Taguchi, Md. Shamim Hossain, Miki Ohira, Yohko Nakamura, Akira Nakagawara

**Affiliations:** 1 Children’s Cancer Research Center, Chiba Cancer Center Research Institute, Chiba 260-8717, Japan; 2 Department of Molecular Biology and Oncology, Graduate School of Medical and Pharmaceutical Sciences, Chiba University, Chiba 260-8670, Japan; 3 Department of Anatomic Pathology, Graduate School Medical Sciences, Kyushu University, Fukuoka 812-8582, Japan, Chiba 260-8670, Japan; 4 Department of Pediatric Surgery, Graduate School Medical Sciences, Kyushu University, Fukuoka 812-8582, Japan; 5 Division of Cancer Genomics, Chiba Cancer Center Research Institute, Chiba 260-8717, Japan; 6 Division of Biochemistry & Innovative Cancer Therapeutics, Chiba Cancer Center Research Institute, Chiba 260-8717, Japan; 7 Saga Medical Centre KOSEIKAN, 400 Nakabaru, Kase-machi, Saga 840-8571, Japan

## Abstract

In neuroblastoma (NB), one of the most common paediatric solid tumours, activation of anaplastic lymphoma kinase (ALK) is often associated with poor outcomes. Although genetic studies have identified copy number alteration and nonsynonymous mutations of *ALK*, the regulatory mechanism of ALK signalling at protein levels is largely elusive. Neuronal leucine-rich repeat 1 (NLRR1) is a type 1 transmembrane protein that is highly expressed in unfavourable NB and potentially influences receptor tyrosine kinase signalling. Here, we showed that NLRR1 and ALK exhibited a mutually exclusive expression pattern in primary NB tissues by immunohistochemistry. Moreover, dorsal root ganglia of *Nlrr1*+/+ and *Nlrr1*−/− mice displayed the opposite expression patterns of Nlrr1 and Alk. Of interest, NLRR1 physically interacted with ALK *in vitro* through its extracellular region. Notably, the NLRR1 ectodomain impaired ALK phosphorylation and proliferation of ALK-mutated NB cells. A newly identified cleavage of the NLRR1 ectodomain also supported NLRR1-mediated ALK signal regulation *in trans*. Thus, we conclude that NLRR1 appears to be an extracellular negative regulator of ALK signalling in NB and neuronal development. Our findings may be beneficial to comprehend NB heterogeneity and to develop a novel therapy against unfavourable NB.

Neuroblastoma (NB) is the most common extracranial solid tumour in children with heterogeneous clinical outcomes. Tumours diagnosed before 18 months of age with a triploid DNA karyotype and low grade of metastasis show favourable outcomes even with spontaneous regression, whereas those in older patients with *MYCN* amplification, a diploid DNA karyotype, and frequent metastasis have unfavourable outcomes with a less than 50% overall survival rate^1^. Recent genomic studies have revealed novel prognostic markers and potential molecular targets in unfavourable NB^2^. However, the crucial roles of the gene products in NB, especially their functional interactions regulating cell signals, are largely elusive.

Anaplastic lymphoma kinase (ALK) is a receptor tyrosine kinase (RTK) that is highly expressed in embryonic nervous tissues and adult brains^3^,^4^,^5^,^6^. Although its physiological ligands are still controversial in mammals^7^,^8^,^9^,^10^,^11^,^12^,^13^, *in vivo* studies have implied that ALK contributes to neurogenesis and learning ability^14^,^15^,^16^. In humans, an aberrant status of ALK, such as fusion oncoproteins by chromosomal translocation, nonsynonymous mutations within the kinase domain, and overexpression, is associated with carcinogenesis and poor prognoses in various cancers^17^. In both familial and approximately 6–10% of sporadic NBs, point mutations have been identified in frequent sites at F1174 and R1275^18^,^19^,^20^,^21^. Among the mutations, F1174L is the most frequent and oncogenic, transforming murine fibroblasts and neural crest cells^21^,^22^, potentiating MYCN-driven tumourigenesis *in vivo*^16^,^23^,^24^,^25^, and showing resistance to the first clinically available ALK inhibitor, crizotinib^26^,^27^. In addition, gene amplification of *ALK*, accounting for around 3% of NBs, and its aberrantly truncated form result in constitutive activation of ALK^21^,^28^,^29^,^30^. Furthermore, overexpression of ALK at mRNA and protein levels without genomic alterations is associated with unfavourable outcomes^31^,^32^,^33^. Therefore, understanding the regulatory mechanism of ALK signalling should advance our knowledge concerning the development of NB.

Neuronal leucine-rich repeat 1 (*NLRR1*), one of the members of the neuronal leucine-rich repeat family encoding type I transmembrane proteins, was previously identified as a prognostic factor in high-risk NB^34^. In the gene family, *NLRR1* and *NLRR3* are expressed in unfavourable and favourable NBs, respectively, and the expression pattern of *NLRR2*^34^ is unknown. Our recent studies have revealed that *NLRR1* is a direct transcriptional target of MYCN, and NLRR1 potentiates epidermal growth factor receptor (EGFR) and insulin-like growth factor receptor (IGFR) signals, which *vice versa* initiates *MYCN* transcription^35^,^36^. However, it is still unclear how NLRR1 influences other RTKs in NB.

Here, we examined the functional relationship between NLRR1 and ALK, and found that NLRR1 suppressed ALK signalling through a direct physical interaction. Immunohistochemistry of both human primary NBs and mouse dorsal root ganglia (DRGs) revealed a mutually exclusive expression pattern of NLRR1 and ALK *in vivo*, which also suggested an inhibitory effect of NLRR1 on ALK.

## Results

### NLRR1 and ALK show mutually exclusive expression in human NB

**Figure 1:**
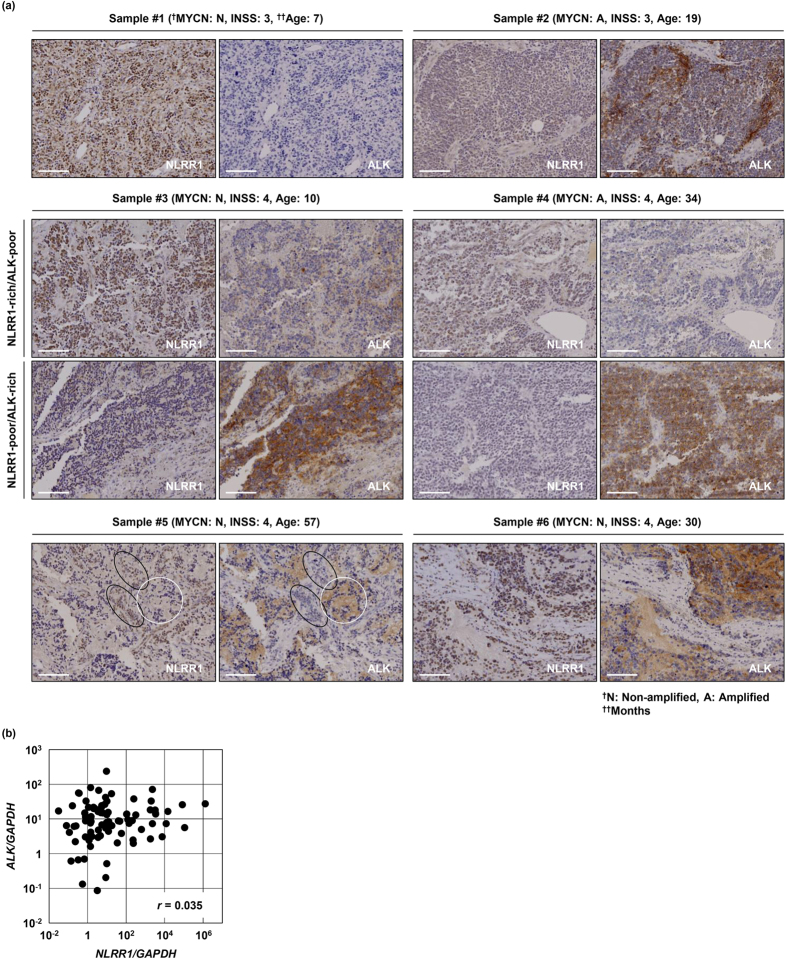
NLRR1 and ALK show mutually exclusive expression in human NB. (**a**) Expression patterns of NLRR1 and ALK were often mutually exclusive in immunohistochemical analysis. Black and white circles indicate NLRR1-rich/ALK-poor and NLRR1-poor/ALK-rich cancer cell clusters, respectively. Bars: 100 μm. (**b**) The expression level of *NLRR1* was not correlated with that of *ALK* in qPCR analysis. The correlation was evaluated by Pearson’s test.

The expression levels of NLRR1 and ALK are associated with unfavourable outcomes of NB^31^,^32^,^33^,^34^. Hence, we randomly selected six primary tumour samples, which were diagnosed as stage 3 or 4, and then immunohistochemically investigated the expression patterns of NLRR1 and ALK in the specimens. Sample #1 with high expression of NLRR1 was negative for ALK, whereas sample #2 with high expression of ALK exhibited low expression of NLRR1 (Fig. 1a). Notably, three specimens showed intratumoural heterogeneity of NLRR1 and ALK. In samples #3 and #4, NLRR1-rich loci were faintly stained for ALK (Fig. 1a, upper panels of samples #3 and #4), while NLRR1-poor regions displayed higher expression of ALK (Fig. 1a, lower panels of samples #3 and #4). Of interest, NLRR1-rich/ALK-poor and NLRR1-poor/ALK-rich cancer cell clusters were mosaically localised in sample #5 (black and white circles in Fig. 1a, respectively). Although sample #6 did not show the same pattern, NLRR1 and ALK were generally expressed in different cancer cells that displayed separated or neighboured clusters even in an identical tumour. To confirm the heterogeneous expression patterns of NLRR1 and ALK, we stained a commercially available human tissue array including 10 NBs. The results are summarised in Supplementary Table 1. Of the tissue array samples, two tumours with high expression of NLRR1 were indistinctly stained for ALK (Supplementary Figure 1a i–iv). Conversely, three tumours with high expression of ALK rarely harboured NLRR1 (Supplementary Figure 1a vii–x), although one section expressed intermediate levels of NLRR1 and ALK (Supplementary Figure 1a v and vi). Therefore, NLRR1 and ALK tended to be oppositely expressed among different tumours. To reveal the expressional correlation at the mRNA level, we performed qPCR analysis using 87 human NB cDNA samples including all International Neuroblastoma Staging System (INSS) stages with *MYCN*-amplified and non-amplified tumours. Despite the opposite expression pattern of NLRR1 and ALK in immunohistochemical analyses, *NLRR1* and *ALK* showed no significant correlation (Pearson’s *r* = 0.035, *P* = 0.749, Fig. 1b and Supplementary Table 2). Furthermore, in risk factor classifications (*MYCN* status, INSS, age, histology, primary tumour site, TrkA expression, and prognosis), the expression level of *NLRR1* was not associated with that of *ALK* (Supplementary Table 2). Because a large tumour sample often included both NLRR1-rich/ALK-poor and NLRR1-poor/ALK-rich cancer cells (Fig. 1a, samples #3, #4, and #5), the opposite expression pattern of NLRR1 and ALK might be masked in the homogenised mRNA samples. We further examined the expression pattern of NLRR1 and ALK in human NB cell lines SK-N-BE, SK-N-DZ, CHP134, SMS-SAN, SH-SY5Y, Kelly, and NB-39-nu. Consequently, in both qPCR and western blot analyses, CHP134 and SMS-SAN cells highly expressed NLRR1, but they exhibited relatively low expression of ALK (Supplementary Figure 1b). The expression status might result in no phosphorylation of ALK in SMS-SAN cells that harbour the F1174L mutation. In contrast, other ALK-mutated cell lines, SH-SY5Y (F1174L), Kelly (F1174L), and NB-39-nu (amplified), highly expressed ALK with constitutive activation, but they showed low expression of NLRR1 (Supplementary Figure 1b). The homogenous properties of cultured cell lines might result in the opposite expression patterns of *NLRR1* and *ALK* at the mRNA level, which were not confirmed in clinical samples, although they exhibited no statistical significance. Taken together, NLRR1 and ALK were generally expressed in different populations of NB cells and exhibited mutually exclusive expression patterns mainly in immunohistochemical analyses.

### NLRR1 and ALK show mutually exclusive expression in murine DRGs

**Figure 2:**
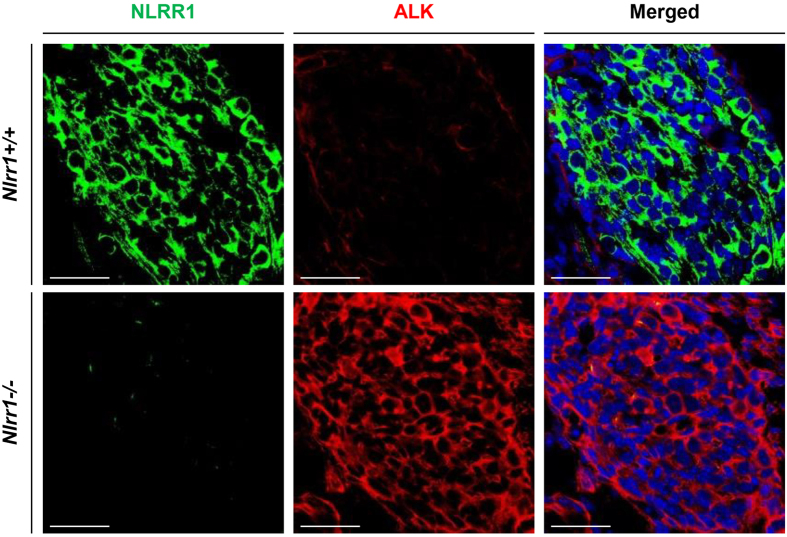
NLRR1 and ALK show mutually exclusive expression in murine DRGs. Nlrr1-expressing cells were enriched in *Nlrr1*+/+ DRGs, whereas ALK-expressing cells occupied DRGs in *Nlrr1−/−* mice. Bars: 50 μm.

Next, we hypothesised opposite expression patterns of NLRR1 and ALK during neuronal development, because both membrane proteins are expressed in embryonic central and peripheral nervous tissues^3^,^4^,^5^,^6^,^37^,^38^. Using *Nlrr1−/−* mouse embryos, we performed immunohistochemistry of Nlrr1 and Alk, and focused on DRGs that originate from neural crest cells, similar to NB. On embryonic day (E) 13.5, Alk was intensely stained in *Nlrr1−/−* DRGs, while the expression level of Alk was low in *Nlrr1*+/+ DRGs, implying that Nlrr1 and Alk were also oppositely expressed during neuronal development (Fig. 2).

### The extracellular region of NLRR1 physically interacts with ALK

**Figure 3:**
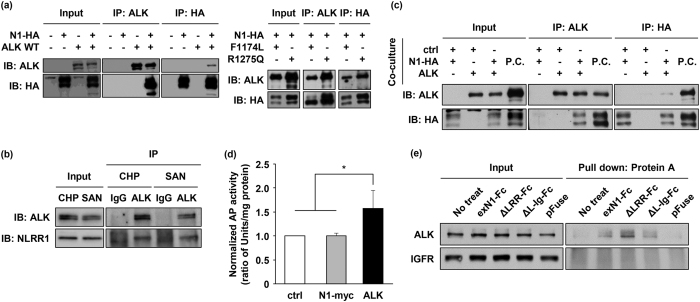
The extracellular region of NLRR1 suppresses ALK via a physical interaction. (**a**) NLRR1 and ALK interacted with each other in co-transfected HEK293 cells. (**b**) The endogenous interaction between NLRR1 and ALK was confirmed in CHP134 and SMS-SAN cells. (**c**) NLRR1 and ALK displayed the physical interaction among neighbouring cells. The positive control (P.C.) was a co-transfected sample. (**d**) exN1 bound to ALK-expressing cells. Data are means ± s.d. *P < 0.05. (**e**) exN1 bound to ALK through the FNIII domain. Uncropped blots are shown in Supplementary Figure 6. The gels were run under the same experimental conditions.

The mutually exclusive expression of NLRR1 and ALK in human NB and murine DRGs encouraged us to investigate their extracellular interaction. First, we transiently expressed NLRR1 tagged with HA (NLRR1-HA or N1-HA) and wild-type (WT) ALK in HEK293 cells and then performed immunoprecipitation followed by western blotting. The results indicated a physical interaction between these membrane proteins (Fig. 3a, left panel). NLRR1 also directly bound to oncogenic ALK mutants F1174L and R1275Q in the transiently co-expressing cells (Fig. 3a, right panel). Treatment of the cells with tunicamycin, an N-liked glycosylation inhibitor, showed no influence on the interaction, suggesting that NLRR1 physically interacts with ALK regardless of its N-glycosylation (Supplementary Figure 2a). The endogenous interaction between NLRR1 and ALKs was confirmed in CHP134 and SMS-SAN cells, both of which endogenously harboured detectable levels of NLRR1 and ALK in western blot analyses (Fig. 3b and Supplementary Figure 1b). Next, we transfected Cos7 cells with NLRR1-HA or WT ALK independently, and then co-cultured them. In the post co-cultured sample, ALK was co-precipitated with NLRR1, suggesting that NLRR1 interacts with ALK through its extracellular region among neighbouring cells (Fig. 3c). To confirm binding of the NLRR1 extracellular domain to ALK, we generated an intact extracellular domain of NLRR1 (exN1) fused with human placental alkaline phosphatase (AP) at the C-terminal (exN1-AP) using a pSecTag2 template vector. Then, Cos7 cells transiently expressing ALK were treated with conditioned medium containing exN1-AP, followed by measurement of AP activities in the lysates. Significantly higher AP activity in ALK-expressing cells compared with control cells indicated binding of the NLRR1 ectodomain to ALK (Fig. 3d). We also investigated potential binding of the NLRR1 ectodomain to NLRR1-expressing cells, because LRR domain has been reported to be responsible for protein dimerlisation^39^. However, the AP activity of NLRR1-expressing cells was comparable to that of the control (Fig. 3d). Additionally, to examine the direct interaction between the NLRR1 ectodomain and ALK, and to determine the domain of NLRR1 responsible for the binding, we newly generated exN1 and its deletion mutants conjugated with human IgG_1_ Fc at the C-terminus using a pFUSE-hIgG1-Fc2 template vector. Because NLRR1 has three domains in the extracellular region, LRR, immunoglobulin-like (Ig-like), and fibronectin type III (FNIII) domains, the following three chimeric proteins were established: exN1-Fc, ΔLRR-FC encompassing Ig-like and FNIII domains, and ΔL-Ig-FC encompassing only the FNIII domain (Supplementary Figure 2b). Human IgG_1_ Fc was used as a negative control. NB-39-nu cells were treated with conditioned medium containing the Fc chimeric proteins, and the lysates were subjected to protein A pull-down. As a result, ALK was co-precipitated with all of the Fc-fused mutants, but not with the Fc control (Fig. 3e), implying that the NLRR1 ectodomain certainly binds to ALK and that the FNIII domain is responsible for the physical interaction between NLRR1 and ALK. No positive band of IGFR in the protein A pull-down indicated specific binding of the NLRR1 ectodomain to ALK (Fig. 3e).

### ALK phosphorylation is suppressed by the extracellular region of NLRR1

**Figure 4:**
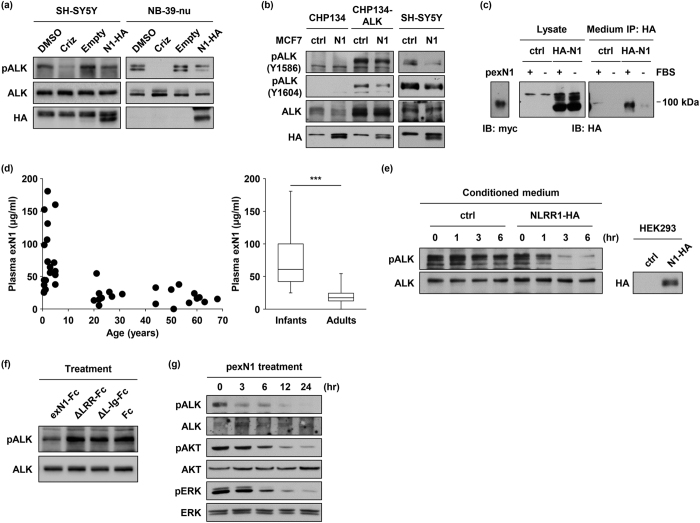
The extracellular region of NLRR1 suppresses ALK phosphorylation. (**a**) Transient expression of NLRR1 suppressed ALK phosphorylation in ALK-mutated NB cell lines. (**b**) ALK phosphorylation was impaired by co-culture with NLRR1-expressing cells. (**c**) The NLRR1 ectodomain was secreted into the culture medium. (**d**) The NLRR1 ectodomain was enriched in human infant plasma. The statistical difference between infants and adults was evaluated by the Mann–Whitney test. ***P < 0.001. (**e**) Conditioned medium of NLRR1-expressing cells repressed ALK phosphorylation. (**f**) The LRR domain was essential for the inhibitory effect on ALK phosphorylation. (**g**) pexN1 treatment attenuated ALK phosphorylation.

Because NLRR1 potentially affects other RTK signals^36^, we examined the ALK phosphorylation status in the presence of NLRR1. We transiently expressed NLRR1-HA in NB cell lines with constitutively activated ALK signalling and a low level of endogenous NLRR1. As the positive control of impaired ALK phosphorylation, SH-SY5Y and NB-39-nu cells were treated with 100 nM crizotinib, a kinase inhibitor of ALK. Although the treatment completely suppressed phosphorylation of amplified ALK, the F1174L mutant was still activated (Fig. 4a). This result was consistent with previous reports in which the F1174L mutant was resistant to crizotinib^26^,^27^. Intriguingly, in both cell lines, NLRR1 overexpression impaired ALK phosphorylation with no obvious alteration of total ALK protein (Fig. 4a). In addition, CHP134 and SMS-SAN cells were subjected to knockdown of NLRR1. Consistent with the reduced ALK phosphorylation upon ectopic NLRR1 expression (Fig. 4a), three independent shRNAs slightly elevated phosphorylation of ALK (Supplementary Figure 3a). Our binding assays revealed an interaction between the NLRR1 ectodomain and ALK (Fig. 3). Hence, the negative effect of NLRR1 on ALK phosphorylation was also examined *in trans*. We co-cultured NB cell lines stably expressing enhanced green fluorescent protein (EGFP) with MCF7 cells transfected with a control vector or NLRR1-HA (MCF7-ctrl and MCF7-NLRR1, respectively), because MCF7 cells had no detectable level of endogenous ALK. Although CHP134 cells harboured no phosphorylated ALK, ectopic ALK expression in the cells (CHP134-ALK) resulted in detectable level of ALK phosphorylation (Fig. 4b). Of note, ectopic and endogenous ALK phosphorylation in CHP134 and SH-SY5Y cells, respectively, were impaired by co-culture with MCF7-NLRR1 cells (Fig. 4b). Consistently, ERK phosphorylation was suppressed in CHP134-ALK cells neighbour on MCF7-NLRR1 cells (Supplementary Figure 3b). Taken together, these results suggested that NLRR1 might attenuate ALK phosphorylation in neighbouring cells.

Considering the separation of NLRR1-rich and ALK-rich cancer cell clusters in human NB tissue specimens, the intracellular short tail of NLRR1, and the proteolytic cleavage of NLRR3 at the transmembrane region^40^, we hypothesised that NLRR1 should also act as a soluble form of the extracellular domain after cleavage. To identify the cleaved form, N-terminal HA-tagged NLRR1 (HA-N1) was expressed in HEK293 cells, and the conditioned medium was subjected to immunoprecipitation against HA. The cleaved N-terminal fragment of NLRR1 was observed in western blot analysis when the cells were cultured in 10% fetal bovine serum (FBS)-containing medium (Fig. 4c). To estimate the molecular weight, we yielded the exN1 protein conjugated with a myc/His tag using a pSecTag2 construct. Purified by His tag affinity columns (pexN1), the pexN1 exhibited almost 90 kDa in western blotting, which was comparable to the cleaved N-terminal fragment of NLRR1 (Fig. 4c). Thus, the intact NLRR1 ectodomain was certainly cleaved and secreted into the culture medium. The N-terminal fragment was decreased under the serum-free condition, suggesting that soluble factors in the serum directly trigger the cleavage or it is affected by cell viability. To test the latter hypothesis, we investigated the cleavage of NLRR1 at different confluences of SH-SY5Y cells transfected with HA-N1. An immunoprecipitation assay employing the conditioned medium showed that shedding of the NLRR1 ectodomain was promoted in a higher confluent condition (Supplementary Figure 3c). Consistent with the enhanced shedding, the cells at higher confluency harboured a lower level of full-length HA-N1 in the total cell lysates. The band intensity of the NLRR1 ectodomain appeared to increase exponentially against the number of cells (Supplementary Figure 3d), suggesting that the shedding may depend not only on the number of cells but also cell-cell contact. Moreover, exN1-containing medium treatment promoted the cleavage (Supplementary Figure 3c,d). This result may shed a light on the reason why NLRR1-expressing cells displayed the low AP activity upon exN1-AP treatment (Fig. 3d). Because the extracellular NLRR1-NLRR1 interaction might trigger shedding of NLRR1, the exN1-AP treatment possibly enhanced the cleavage and release of full-length NLRR1 from the cell surface, thereby leading to the low AP activity after washing. Taken together, the extracellular interaction of full-length NLRR1 proteins should trigger their own cleavage. Furthermore, to examine the presence of the NLRR1 ectodomain *in vivo*, we performed immunoprecipitation analysis employing healthy human plasma samples. As a result, the NLRR1 ectodomain was detected in three infant plasma samples, but not in adult plasma samples (Supplementary Figure 3e). Moreover, we developed a sandwich enzyme-linked immunosorbent assay (ELISA) system to measure the concentration of the NLRR1 ectodomain in human plasma. Consistent with the immunoprecipitation, the concentration of the NLRR1 ectodomain in healthy infant (<10 years old) plasma was significantly much higher than that in adult (>20 years old) samples (median value: 60.8 μg/ml vs. 17.3 μg/ml, Fig. 4d). These data indicated the presence of the soluble NLRR1 ectodomain *in vivo*, especially in children.

To further assess the inhibitory effect of the NLRR1 ectodomain on ALK, we treated SH-SY5Y cells with conditioned medium of HEK293 cells expressing full-length NLRR1. As expected, the medium inhibited ALK phosphorylation in a time-dependent manner (Fig. 4e). We next treated SH-SY5Y cells with the conditioned medium of HEK293 cells including Fc-chimeric exN1 and its deletion mutants. Consequently, exN1-Fc impaired the phosphorylation of ALK, whereas ΔLRR-FC and ΔL-Ig-FC had no effect on the signal (Fig. 4f). These data suggested the importance of the LRR domain or full-length structure of NLRR1 in the inhibitory effect on ALK phosphorylation although the FNIII domain was shown to be responsible for the binding (Fig. 3e). Therefore, the functioning and binding domains of NLRR1 are possibly different. Furthermore, pexN1 treatment impeded the phosphorylation of ALK, AKT, and ERK in SH-SY5Y cells (Fig. 4g). Therefore, we concluded that the NLRR1 ectodomain negatively regulated ALK signalling *in trans*.

### NLRR1 impairs ALK-expressing cell expansion

**Figure 5:**
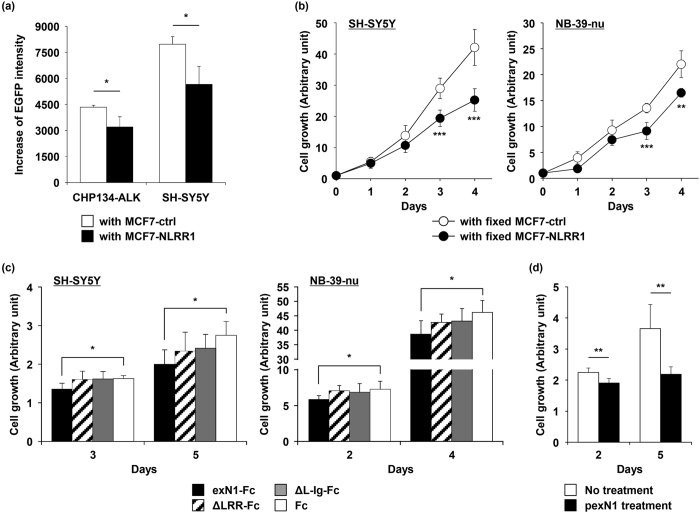
NLRR1 impairs ALK-expressing cell expansion. (**a**) ALK-expressing NB cell expansion was repressed by co-culture with NLRR1-expressing cells. Cell expansion for 5 days was evaluated as the increase of EGFP intensity. Data are means ± s.d. (**b**) ALK-mutated NB cell growth was inhibited by a fixed layer of NLRR1-expressing cells. (**d**) exN1 abrogated ALK-mutated NB cell proliferation and the LRR domain was required for the effect. (**d**) pexN1 reduced SH-SY5Y cell growth. Data are means ± s.d. *P < 0.05, **P < 0.01, and ***P < 0.001.

Considering the negative effect of the NLRR1 ectodomain on ALK signalling, we hypothesised that the proliferation of ALK-mutated NB cells might be suppressed by the NLRR1 ectodomain. In the co-culture assay performed in Fig. 4b, the increase of total EGFP intensity in CHP134-ALK and SH-SY5Y cells was significantly diminished by the neighbouring MCF7-NLRR1 cells (Fig. 5a and Supplementary Figure 4a). The live MCF7-NLRR1 cells possibly occupied a larger area than the control cells, resulting in suppression of NB cell growth in the co-culture condition. Hence, we next fixed MCF7-NLRR1 cells on the surface of the culture plate and then incubated SH-SY5Y and NB-39-nu cells in the wells. Consequently, the cell proliferation was remarkably attenuated by culture on the fixed NLRR1-expressing cell layer compared with that on the fixed control cells (Fig. 5b). To evaluate the inhibitory effect on cell growth by the exN1 molecule, Fc-chimeric exN1 and its deletion mutants were introduced into 96-well plates coated with protein G. Then, SH-SY5Y and NB-39-nu cells were cultured in these plates. Of the chimeric proteins, fixed exN1-Fc suppressed cell proliferation, whereas the other two deletion mutants exhibited marginal effects (Fig. 5c). This result also suggested that the negative effect of NLRR1 on ALK required the LRR domain. Furthermore, pexN1 treatment decreased the growth of SH-SY5Y cells compared with untreated and vehicle-treated controls (Fig. 5d and Supplementary Figure 4b). Therefore, the NLRR1 ectodomain certainly suppressed ALK-mutated NB cell proliferation.

## Discussion

Here, we provide the first evidence that NLRR1 and ALK exhibit mutually exclusive expression patterns in NB tissues and murine DRGs, and that NLRR1 binds to ALK through its extracellular region to impair ALK functions in cell proliferation. Several ligands have been reported to activate EGFR signalling^41^, whereas an extracellular antagonist against human EGFR has been identified recently^42^. These reports imply the complexity of ALK signal regulation by potential ALK ligands^7^,^8^,^12^,^13^ and unknown extracellular antagonists. Our present data strongly suggest that NLRR1 appears to be the first extracellular antagonist against ALK signalling, although this finding needs to be further confirmed. Intriguingly, NLRR1 undergoes proteolytic cleavage to release its ectodomain, which in turn binds to ALK to inhibit its functions. Although the precise mechanism of NLRR1 shedding is unknown, the fact that a protease inhibitor cocktail blocked the shedding implies protease(s)-dependent cleavage (Supplementary Figure 5).

The functioning and binding domains of NLRR1 are possibly different, because the FNIII domain alone was co-precipitated with ALK (Fig. 3e), whereas depletion of the LRR domain lacked the inhibitory effect on ALK phosphorylation (Fig. 4f). According to a previous report, the LRR domain is responsible for protein dimerisation and ligand-receptor interaction^39^. In addition, our data suggested that dimerisation of NLRR1 triggers shedding of the NLRR1 ectodomain (Supplementary Figure 3c,d), and the cleaved ectodomain suppressed ALK phosphorylation (Fig. 4e–g). Hence, we hypothesised that LRR domain-mediated dimerisation of NLRR1 might have an important role in the release of the ectodomain and abrogation of ALK functions.

We previously reported that MYCN transcriptionally targets *NLRR1*^35^ to enhance NB cell growth, and that NLRR1, *vice versa*, positively regulates MYCN via EGFR signal enhancement^36^. Conversely, MYCN also transactivates *ALK*^43^, and ALK signalling promotes *MYCN* transcription through activation of ERK5^44^. Thus, both NLRR1 and ALK form positive feedback loops with MYCN. However, these MYCN-associated regulatory mechanisms do not exist simultaneously in a single cell under physiological conditions. That is because NLRR1 and ALK were independently expressed in separated cell clusters of an identical human NB biopsy (Fig. 1a), and the NLRR1 ectodomain had a negative effect on ALK. Therefore, we believe that NLRR1-mediated regulation occurs in neighbouring or separated NB cells, which controls the ALK-expressing cell population and in part contributes to the mutually exclusive expression of NLRR1 and ALK. Specifically, ALK-rich cells may not expand into NLRR1-rich cell clusters, but they proliferate well in an NLRR1-poor environment. Accordingly, NLRR1 is a direct negative regulator of ALK in *trans*, but an indirect positive regulator of EGFR in *cis*, although both ALK and EGFR signals enhance cell survival and proliferation.

The physiological and pathological roles of ALK signal regulation by NLRR1 during neuronal development and carcinogenesis remain unclear. We found that some cancer cells were double-positive for both proteins (sample #6 in Fig. 1a and sample #3 in Supplementary Figure 1). Currently, the biological and clinical significance of double-positive, NLRR1-rich/ALK-poor, and NLRR1-poor/ALK-rich cancer cells remain to be uncovered. However, intratumoural heterogeneity is one of the crucial problems encountered when treating patients with cancer including high-risk NB. Our observation of the mutually exclusive expression of NLRR1 and ALK in normal and cancerous tissues might provide some important insights into the gene regulation of heterogeneous patterning within these tissues. Thus, targeted therapy against both NLRR1 and ALK might be possible to treat patients with particular cancers such as high-risk NB.

## Methods

### Immunohistochemistry of NB tissues

The six NB specimens were surgically collected and embedded in paraffin at Kyushu University Hospital. The appropriate informed consent was obtained under the institutional review board-approved protocol. The formalin-fixed, paraffin-embedded specimens and a purchased tissue array MC803a (U.S. Biomax) were deparaffinised and stained with an anti-hNLRR1 antibody (R&D, 1:250) overnight. The signal was detected by a biotin-conjugated secondary antibody and streptavidin-horseradish peroxidase (HRP) (VECTOR), followed by diaminobenzidine staining (VECTOR). An ALK detection kit (Nichirei) was used according to the manufacturer’s protocol. The study was approved by the Kyushu University of Medical Human Investigation Committee and the internal review board of the Chiba Cancer Center. The study was performed in accordance with the approved guideline.

### Real-time qPCR

Total RNA was extracted by the acid guanidinium thiocyanate-phenol-chloroform method from clinical NB samples kindly donated by various institutions and hospitals in Japan to the Chiba Cancer Center Neuroblastoma Tissue Bank. Reverse transcription was performed with SuperScript II reverse transcriptase (Invitrogen). qPCR was carried out using the 7500 Real-Time PCR System (Applied Biosystems) according to the manufacturer’s protocol. We employed TaqMan probes for *NLRR1* (Assay ID: Hs00979743_m1) and *ALK* (Assay ID: Hs00608292_m1). The gene expression was standardised to glyceraldehyde-3-phosphate dehydrogenase (*GAPDH*) mRNA levels. This study procedure was approved by the internal review board of Chiba Cancer Center and appropriate informed consent was obtained from each institution or hospital. The study was demonstrated in accordance with approved guidelines. To quantify *NLRR1* and *ALK* expression in NB cell lines, total RNA was extracted by Trizol from the indicated cell lines according to the manufacture’s protocol. Reverse transcription was performed with SuperScript II reverse transcriptase, and the PCR was performed in the 7500 Real-Time PCR System using SYBR Premix Ex Taq (Takara). The specific primer sequences were: NLRR1, 5′-GCAGCTTTTCAACTTGACTGAA-3′ and 5′-TGAAGGTTGCTGAGGTCTTGT-3′; ALK, 5′-CACTCCAGGGAAGCATGG-3′ and 5′-CACTCGAAATGGGTTGTCTG-3′; GAPDH, 5′- ACCTGACCTGCCGTCTAGAA-3′ and 5′-TCCACCACCCTGTTGCTGTA-3′.

### Immunohistochemistry of mouse embryos.

*Nlrr1*+/+ and *−/−* mouse embryos at E13.5 were fixed with 4% paraformaldehyde in PBS at 4 °C and frozen in O.T.C. compound (Sakura). Transverse sections were incubated with anti-mNlrr1 (raised against the C-terminal of mouse Nlrr1, 1:1,000) and anti-ALK (DAKO, 1:50) antibodies overnight at 4 °C. A M.O.M. Immunodetection Kit (VECTOR) was used to stain with antibodies raised in mice. The primary antibodies were labelled by secondary anti-rabbit IgG Alexa 488 (Molecular Probes, 1:500) and biotinylated anti-mouse IgG (1:250), followed by avidin Cy3 (1:1,000). The mouse experiments were approved by the Committee for Animal Care at the Chiba Cancer Center Research Institute and carried out in accordance with approved guidelines and regulations

### Cell culture and transfection

NB cell lines (SK-N-BE, SK-N-DZ, CHP134, SMA-SAN, SH-SY5Y, Kelly, and NB-39-nu) and non-NB cell lines (HEK293, MCF7, and Cos7) were cultured in RPMI-1640 or Dulbecco’s modified Eagle’s medium (Sigma) supplemented with 10% FBS and 1% penicillin/streptomycin (Gibco). Cells were transfected using FuGENE HD (Promega) or Lipofectamine 3000 (Life technologies) according to the manufacturers’ instructions. EGFP-expressing stable clones were selected and maintained in medium containing 400 μg/ml G418 (Sigma).

### Plasmids

Human *NLRR1* (NM_020873) tagged with HA at the C-terminal was cloned into the BamHI/NotI site of pcDNA3.1-myc-His (Invitrogen). This construct was used to generate other NLRR1-related plasmids. For secretion of the intact NLRR1 ectodomain, the corresponding PCR-amplified fragment of the intact extracellular region (exN1: M1-L632) was inserted into the BamHI/XhoI site of the pSecTag2B vector (Invitrogen). To fuse exN1 with *human placental alkaline phosphatase (AP*: NM_001632), the PCR fragment of AP (I18-G489) were inserted into the C-terminal of the pSecTag2B-exN1 vector using the In-Fusion cloning technique (Clontech). To generate the Fc chimeric proteins, intact exN1 and its deletion mutants (exN1-Fc: M1-L632; ΔLRR-Fc: N362-L632; ΔL-Ig-Fc: V516-L632) were introduced into the EcoRI site of pFUSE-hIgG1-Fc2 (Invivogen). The pEGFP-N1 plasmid was employed for EGFP-expressing cells. Expression vectors for WT ALK and ALK F1174L were kind gifts from Dr. Junko Takita (The University of Tokyo, Tokyo, Japan). The ALK R1275Q construct was generated using site-directed mutagenesis. Mission short hairpin RNA constructs, NM_020873.3-427s1c1, NM_020873.3-846s1c1, and NM_020873.3-1359s1c1, were purchased from Sigma.

### Immunoprecipitation

All procedures were performed at 4 °C. Cells were lysed in CHAPS lysis buffer [50 mM Tris-HCl, pH 7.4, 100 mM NaCl, 10 mM MgCl_2_, 1 mM EDTA, 10 mM CHAPS, and 1 × Protease Inhibitor cocktail (Roche)]. The lysates (500–2,000 μg protein) and 10 ml conditioned medium were precleared with 20 μl protein A agarose bead (Invitrogen) 50% slurry in 500 μl IP buffer (50 mM Tris-HCl, pH 7.4, 150 mM NaCl, 1% Nonidet P-40, and 1 × Protease Inhibitor cocktail) for 30 minutes. The precleared samples were then incubated with anti-HA tag (Roche), anti-ALK (Cell Signaling Technology), and anti-NLRR1 (prepared in-house or purchased from R&D Systems) antibodies for 1 hour, followed by addition of 20 μl protein A agarose bead 50% slurry. Normal rabbit IgG (Santa Cruz Biotechnology) was employed for the negative control. After overnight rotation, the beads were washed six times with 500 μl IP buffer (rotated for 10 minutes twice and inverted four times) and then subjected to western blotting.

### Western blotting

The lysates (25–100 μg protein) in CHAPS or RIPA buffer (20 mM Tris-HCl, pH 7.6, 140 mM NaCl, 1% Nonidet P-40, 0.5% sodium deoxycholate, 0.1% SDS, and 1× Phosphatase Inhibitor cocktail) and precipitated protein A agarose beads were boiled in sample buffer for 5 minutes and applied to polyacrylamide gels. After separation and transfer, the membranes were reacted with primary antibodies overnight at 4 °C. The primary antibodies were anti-pALK Y1586 and 1604, anti-pAKT, anti-AKT, anti-pERK, anti-ERK, anti-myc tag (Cell Signaling Technology), anti-ALK (Beckman), anti-NLRR1 (prepared in-house), anti-HA (Roche), and anti-hIgG1Fc (Millipore) diluted at 1:1000 in TBST. After incubation with HRP-conjugated secondary antibodies (Santa Cruz Biotechnology, 1:5,000), the protein bands were fluoresced by a chemiluminescent substrate (Thermo Scientific).

### Binding assays

Transfected Cos7 cells were incubated for 1 hour at room temperature in ultrafiltrated medium from HEK293 cells transfected with the exN1-AP construct. After washing and lysis, AP activities in the lysates were measured by Lab Assay ALP (Wako) according to the manufacturer’s instructions. The results were standardised to the control. For the pull-down with protein A, NB-39-nu cells were incubated in conditioned medium containing Fc chimeras from HEK293 cells for 1 hour at 37 °C. The washed samples were lysed in CHAPS buffer and 1 mg of sample was directly reacted with 30 μl protein A agarose bead 50% slurry in IP buffer overnight at 4 °C. The samples were washed and then subjected to western blotting.

### Treatments

To reveal the interaction between NLRR1 and ALK under unglycosylated conditions, HEK293 cells were treated with 5 μg/ml tunicamycin for 8 hours and then subjected to immunoprecipitation. Crizotinib (100 nM) was added to the culture media of SH-SY5Y and NB-39-nu cells for 24 hours as the positive control to suppress phosphorylation of ALK. To induce the negative effect of the NLRR1 ectodomain on ALK phosphorylation, SH-SY5Y cells were exposed to the conditioned medium of HEK293 cells transfected with NLRR1-HA or pFUSE constructs, and 10 μg/ml pexN1. A His-tagged protein purification kit (MBL) was employed to generate pexN1.

### Co-culture assay

For the immunoprecipitation, Cos7 cells transfected with either NLRR1-HA or ALK were co-cultured at the same density for 24 hours and then subjected to immunoprecipitation with a 3,3′-dithiobis(sulfosuccinimidyl propionate) cross-linking reaction. Established EGFP-expressing stable NB cell lines CHP134-ALK and SH-SY5Y were combined with MCF7-ctrl or MCF7-NLRR1 cells at 1 × 10^4^ cells per cell line in 24-well plates. Co-cultured samples were also subjected to western blot analysis and fixed with 4% paraformaldehyde, followed by staining with the anti-pERK antibody.

### Detection of exN1 *in vivo*

Human plasma samples (5 μl) were mixed with 500 μl IP buffer, and the mixtures were pre-cleaned with protein G sepharose beads. exN1 was precipitated with 1 μl sheep anti-hNLRR1 antibody (R&D) and then detected by mixed mouse anti-hNLRR1 antibodies (both antibodies were raised against the N-terminal of human NLRR1, 1:1,000) with a HRP-conjugated secondary antibody. In sandwich ELISA analysis, human plasma samples were captured by the sheep anti-hNLRR1 antibody. The mouse anti-hNLRR1 antibody mixture was used for detection.

### Cell growth assay

To evaluate the effect of the NLRR1 ectodomain on ALK-mutated NB cells, MCF7 cells transfected with NLRR1-HA were seeded in a 96-well plate and grown to 70–80% confluence for 24 hours. The cells were then fixed with −20 °C methanol for 15 minutes. For the cell growth assay on Fc chimeric proteins, protein G-coated 96-well plates were incubated with conditioned medium from HEK293 cells transfected with pFUSE constructs for 1 hour at room temperature. SH-SY5Y and NB-39-nu cells were seeded into the modified plates at 1 × 10^3^ cells per well. SH-SY5Y cells seeded into a normal 96-well plate were also treated with 10 μg/ml pexN1. Cell growth was measured by Cell Counting kit-8 (Dojindo) and a live cell imaging system, Incucyte. The quantified growth was standardised to that on day 0.

### Statistics

The correlation between the expression of *NLRR1* and *ALK* in real-time qPCR analysis was examined by Pearson’s test. The median concentrations of the NLRR1 ectodomain in human infant and adult plasma samples were compared by the Mann-Whitney test. Significance in cell growth analyses was evaluated by the Student’s *t*-test after analysis of variance. The binding assay was tested by the Student’s *t*-test only.

## Additional Information

**How to cite this article**: Satoh, S. *et al.* Neuronal leucine-rich repeat 1 negatively regulates anaplastic lymphoma kinase in neuroblastoma. *Sci. Rep.* **6**, 32682; doi: 10.1038/srep32682 (2016).

## Supplementary Material

Supplementary Information
