# Supplementary material for: Neuronal leucine-rich repeat 1 negatively regulates anaplastic lymphoma kinase in neuroblastoma
Source: Sci Rep. 2016 Sep 8;6:32682. doi: 10.1038/srep32682 (PMC5015029; doi:10.1038/srep32682)
Supplement: Supplementary Information [file srep32682-s1.pdf]

Supplementary Information

Neuronal leucine-rich repeat 1 negatively regulates anaplastic lymphoma kinase in neuroblastoma.

Shunpei Satoh, Atsushi Takatori, Atsushi Ogura, Kenichi Kohashi, Ryota Souzaki, Yoshiaki Kinoshita, Tomoaki Taguchi, Md. Shamim Hossain, Miki Ohira, Yohko Nakamura, Akira Nakagawara

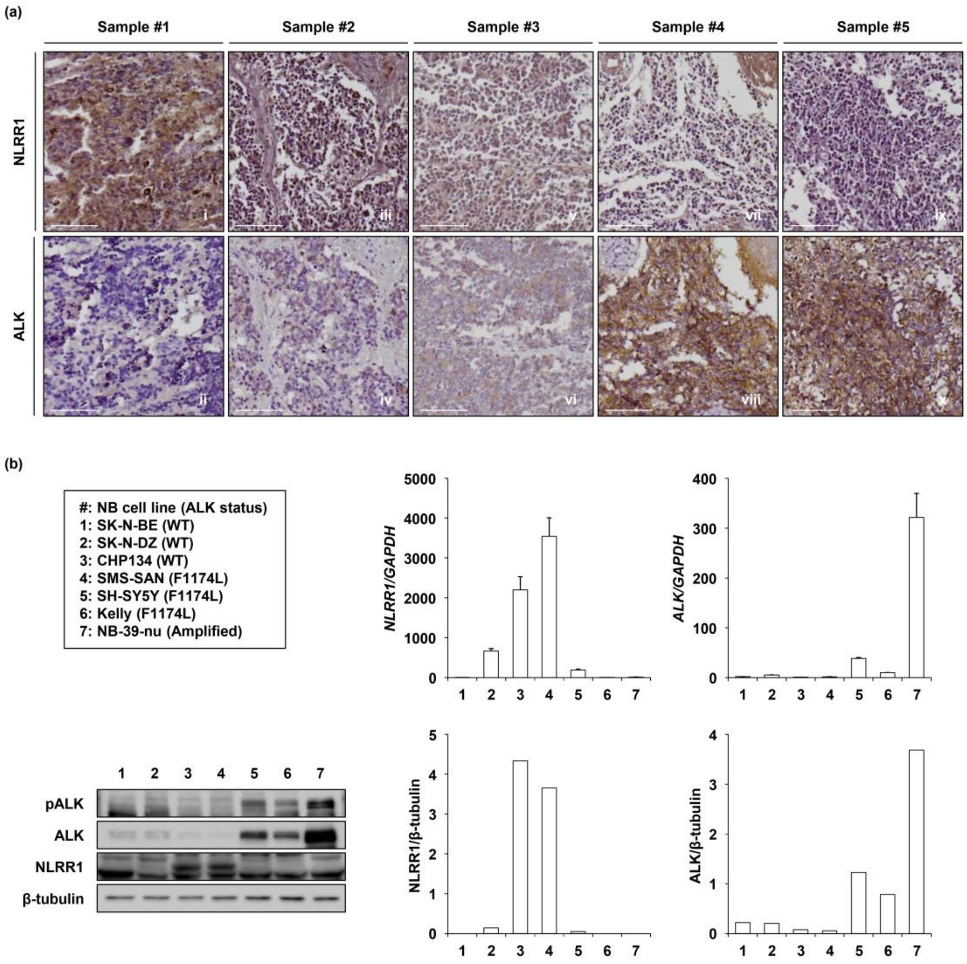

Supplementary Figure 1

a. ALK expression was lower in NLRR1-rich human NB tissues whereas it was enriched in NLRR1-poor ones. Bars: 50 μm. **b**. The expression patterns of NLRR1 and ALK tended to be converse among human NB cell lines.

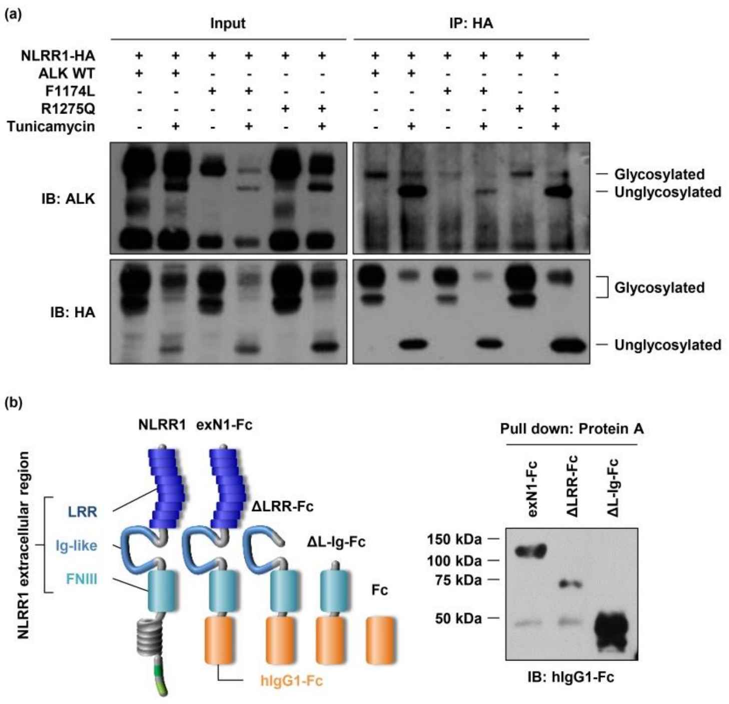

Supplementary Figure 2

a. Unglycosylated NLRR1 and ALK exhibited a physical interaction. **b**. The structure of NLRR1 and the Fc-chimeric proteins were displayed.

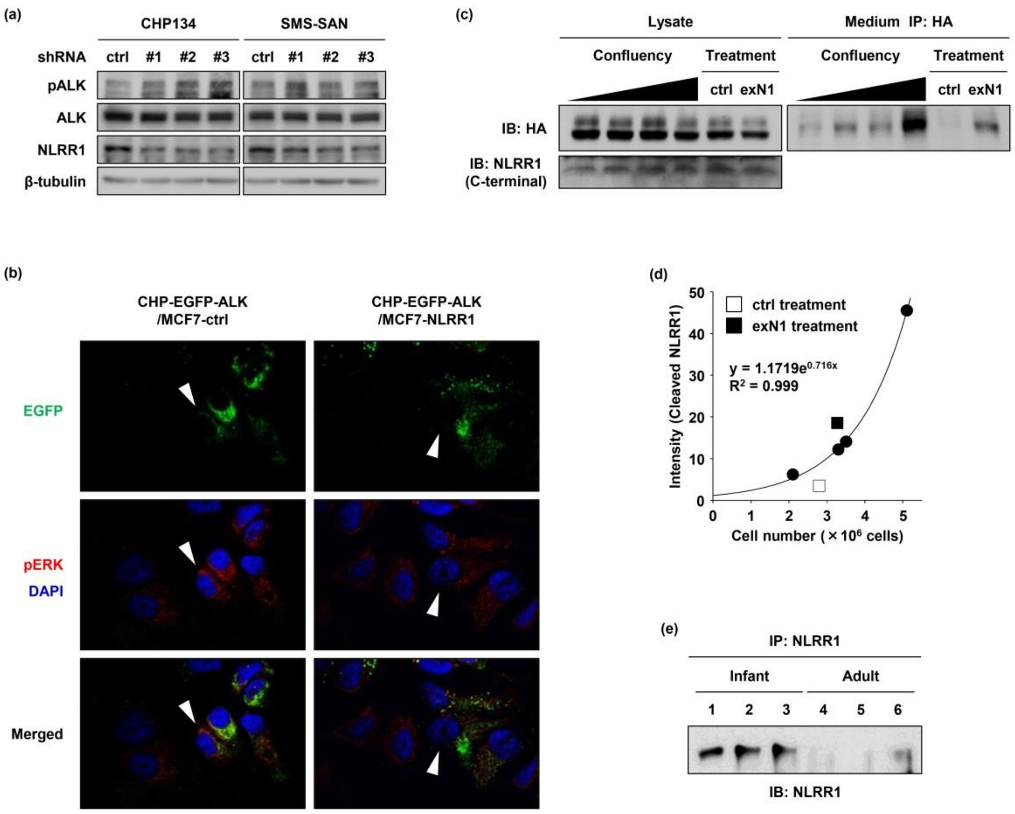

Supplementary Figure 3

a. The knockdown of NLRR1 restored phosphorylation of ALK in CHP134 and SMS-SAN cells. **b**. Phosphorylation of ERK in CHP134-EGFP-ALK was decreased by neighboring MCF7-NLRR1 cells. White arrows indicate CHP134-EGFP-ALK cells. **c, d**. High cellular confluency and the exN1 treatment enhanced the cleavage of the NLRR1 ectodomain. **e**. The NLRR1 ectodomain was highly secreted into human infant plasma.

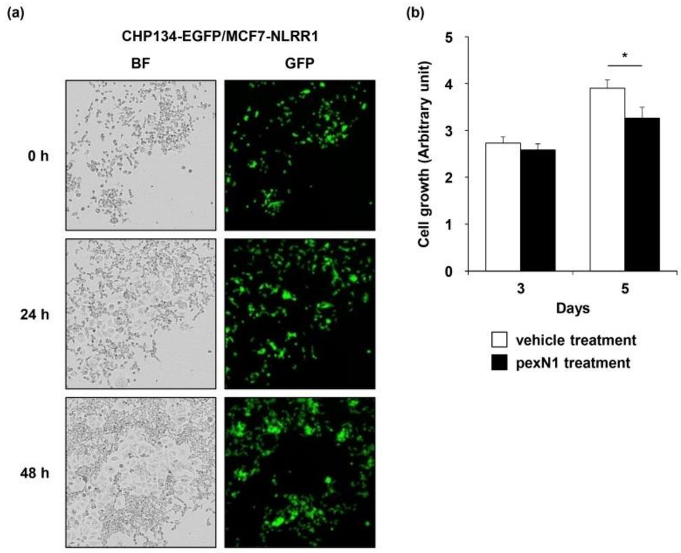

Supplementary Figure 4

a. A live cell imaging system showed the expansion of EGFP fluorescent cells. **b**. pexN1 treatment impaired SH-SY5Y cell proliferation. Through flow of the mock cell conditioned medium in His tag affinity purification was utilized for the control. Data are means ± s.d. *P<0.05

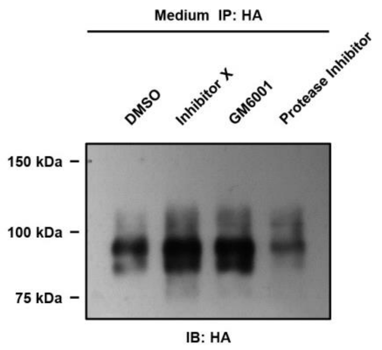

Supplementary Figure 5

Protease inhibitor cocktail reduced the shedding of the NLRR1 ectodomain. The conditioned medium of SH-SY5Y cells transfected with HA-N1 was subjected to immunoprecipitation against HA-tag. A γ-secretase inhibitor, inhibitor X (1.0 μM), a matrix metalloprotease inhibitor, GM6001 (25 μM), and protease inhibitor cocktail were supplemented in the culture medium 6 hours before the immunoprecipitation.

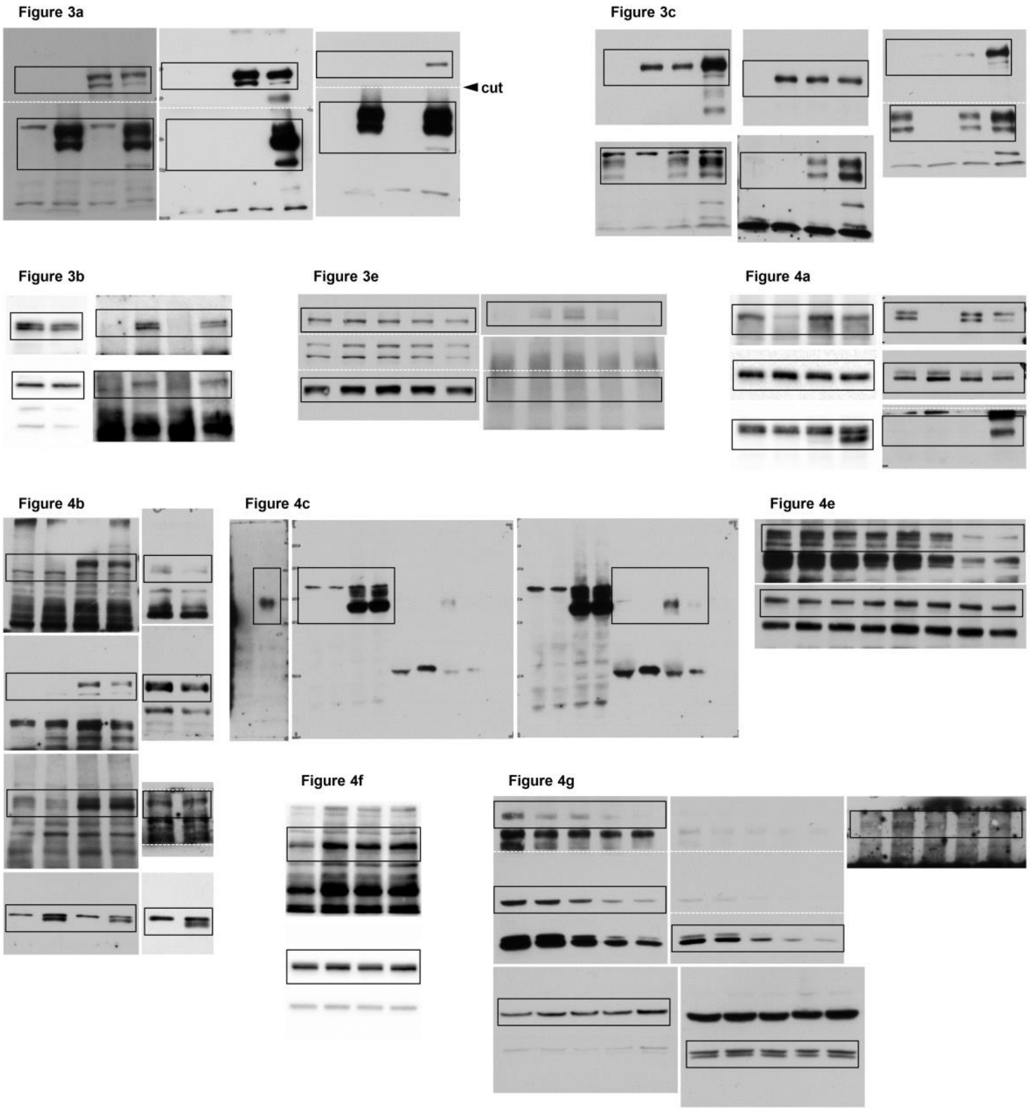

Supplementary Figure 6

Uncropped figures were shown.

Supplementary Table 1

The immunostaining of NLRR1 and ALK in human NB tissue array

Sample	Primary site	NLRR1	ALK
#1	Mediastium	high	-
#2	Abdominal cavity	high	low
#3	Pelvic cavity	Intermediate	Intermediate
#4	Adrenal gland	low	high
#5	Adrenal gland	-	high
#6	Adrenal gland	-	-
#7	Adrenal gland	low	high
#8	Adrenal gland	-	-
#9	Nose	low	-
#10	Nose	-	-

Supplementary Table 2

The expressional correlation of *NLRR1* and *ALK* in human NB

	n	Pearson's *r*	*P*
Whole	87	0.035	0.749
MYCN			
Amplified	73	-0.064	0.589
Non-amplified	14	-0.047	0.872
INSS			
1, 2, 4S	39	-0.086	0.601
3, 4	48	0.021	0.887
Age			
< 18	43	0.004	0.980
≥ 18	44	0.134	0.387
Histology			
Favorable	55	0.070	0.307
Unfavorable	26	0.180	0.190
ND	6		
Primary site			
Adrenal	43	0.147	0.346
Others	44	-0.064	0.678
TrkA			
High	46	-0.075	0.621
Low	39	0.033	0.842
ND	2		
Prognosis			
Alive	63	-0.071	0.582
Dead	24	0.012	0.955
